# Rapid, equipment-free extraction of DNA from skin biopsies for point-of-care diagnostics

**DOI:** 10.1038/s41598-024-64533-3

**Published:** 2024-06-14

**Authors:** Jason Cade Manning, Juan Manuel Boza, Ethel Cesarman, David Erickson

**Affiliations:** 1 Meinig School of Biomedical Engineering, Cornell University, Ithaca, NY 14850 USA; 2 Pathology and Laboratory Medicine, Weill Cornell Medical College, New York, NY 10021 USA; 3 Sibley School of Mechanical and Aerospace Engineering, Cornell University, Ithaca, NY 14850 USA; 4 Division of Nutritional Sciences, Cornell University, Ithaca, NY 14850 USA; 5 Cornell University, 369 Upson Hall, Ithaca, NY 14853 USA

**Keywords:** Alkaline extraction, DNA extraction, Point-of-care, Diagnostics, Kaposi’s sarcoma, Biomedical engineering, Diseases

## Abstract

Kaposi’s sarcoma (KS) is a cancer affecting skin and internal organs for which the Kaposi’s sarcoma associated herpesvirus (KSHV) is a necessary cause. Previous work has pursued KS diagnosis by quantifying KSHV DNA in skin biopsies using a point-of-care (POC) device which performs quantitative loop-mediated isothermal amplification (LAMP). These previous studies revealed that extracting DNA from patient biopsies was the rate limiting step in an otherwise rapid process. In this study, a simplified, POC-compatible alkaline DNA extraction, ColdSHOT, was optimized for 0.75 mm human skin punch biopsies. The optimized ColdSHOT extraction consistently produced 40,000+ copies of DNA per 5 µl reaction from 3 mg samples—a yield comparable to standard spin column extractions—within 1 h without significant equipment. The DNA yield was estimated sufficient for KSHV detection from KS-positive patient biopsies, and the LAMP assay was not affected by non-target tissue in the unpurified samples. Furthermore, the yields achieved via ColdSHOT were robust to sample storage in phosphate-buffered saline (PBS) or Tris-EDTA (TE) buffer prior to DNA extraction, and the DNA sample was stable after extraction. The results presented in this study indicate that the ColdSHOT DNA extraction could be implemented to simplify and accelerate the LAMP-based diagnosis of Kaposi’s sarcoma using submillimeter biopsy samples.

## Introduction

Kaposi’s sarcoma (KS) is a cancer affecting skin and internal organs for which the Kaposi’s sarcoma associated herpesvirus (KSHV) is a necessary cause^1–3^. KS is one of the more common malignancies across sub-Saharan Africa where it leads to high mortality^4–6^. Early detection and treatment with antiretroviral therapy and chemotherapeutics can mitigate the effects of KS, but receiving a histopathological diagnosis is a lengthy process inaccessible outside of centralized clinics^7,8^. Previous work has pursued KS diagnosis by quantifying KSHV DNA in skin biopsies using a point-of-care (POC) device, TINY, which performs quantitative loop-mediated isothermal amplification (LAMP)^9,10^. However, extracting DNA from the patient biopsies adds time and complexity to the otherwise rapid detection of KSHV.

Though rapid DNA extraction methods have been developed for the POC, the methods typically apply to liquid samples and not solid tissues^11,12^. Traditionally, large skin punch biopsies are enzymatically homogenized—a process which sometimes requires overnight incubation—before extracting and purifying DNA^9,13^. While mechanical homogenizers can accelerate the process^14,15^, the required equipment may not be practical for POC applications. To reduce the time and equipment needed, smaller tissue samples may obviate the need for full homogenization. Using a mobile suitcase laboratory, a fast heat and bead-based DNA extraction was used on 2 mm skin biopsies^16^. Additionally, previous work demonstrated the feasibility of using submillimeter skin biopsy samples with the alkaline DNA extraction^17^.

Alkaline extraction uses simple sodium hydroxide and buffering solutions to extract DNA^18,19^. The combination of heat (95 °C) and alkalinity causes hydrolyzation of cellular and nuclear membranes which releases DNA into the solution. While published protocols include heat, NaOH alone is sufficient to hydrolyze cellular membranes^20^, and NaOH is commonly used to decellularize tissues^21–23^. Furthermore, alkaline extraction does not utilize digestive enzymes which require heat denaturation. While heat may increase the rate of DNA extraction and the final yield, a heat-free alkaline extraction could enable equipment-free DNA extraction to simplify and accelerate LAMP-based KSHV detection at the POC.

In this study, the DNA yield from an equipment-free alkaline extraction—ColdSHOT—was optimized using 0.75 mm human skin punch biopsies and LAMP. Once optimized for incubation duration and ratio of tissue sample to final volume, the ColdSHOT extraction was compared to previously established methods including HotSHOT^19^ and a laboratory standard spin column extraction. Finally, several experiments were conducted to assess whether the ColdSHOT extraction could be practically implemented at the POC for the LAMP-based diagnosis of Kaposi’s sarcoma using biopsy samples.

## Methods

### Preparation of skin samples

Residual human skin tissue from surgical specimens after diagnosis was obtained from the Department of Pathology and Laboratory Medicine of Weill Cornell Medicine/The New York Presbyterian Hospital New York City, NY and stored at − 20 °C. Approval was obtained from the Weill Cornell Medicine Institutional Review Board, and the anonymized tissues were used in accordance with the relevant guidelines. From the frozen tissue specimens, submillimeter skin punch biopsies, nominally 0.75 mm in diameter, were collected using a dedicated tool (Electron Microscopy Sciences, 69039-07). To report DNA yield as a function of tissue mass, ten 0.75 mm punch biopsies were weighed and 1.5 mg was used as the average mass of a single 0.75 mm biopsy.

### Real-time LAMP assays

DNA yields reported in this study were quantified via LAMP assays. Briefly, LAMP is an isothermal technique to amplify target sequences with high specificity using 4–6 primers and a strand-displacing DNA polymerase^24^. Self-hybridizing regions in the forward and backward inner primers result in dumbbell structures which serve as seeds for exponential amplification. For POC diagnostics, LAMP is an attractive alternative to polymerase chain reaction (PCR) as LAMP requires only a constant temperature and is known to be more resistant to sample impurities^25,26^.

Two LAMP assays were used in this study—one targeting the human GAPDH sequence^27^ and the other targeting the ORF26 gene of KSHV^28^. The primers for the ORF26 assay were designed to target a conserved region of KSHV and recognize all public variants in the National Center for Biotechnology Information (NCBI) database to date. The primer sets (Supplementary Tables S1 and S2) were obtained from IDT Technologies. The mastermix for each LAMP assay (Supplementary Table S3) was tested against DNA extracted from human skin, ORF26 plasmids^29^, and water to confirm specificity. All real-time LAMP reactions were performed using TINY for 50 min at 68 °C^10^. After preparing the mastermix, 5 µl of sample was added, and all tubes were topped with 20 µl of mineral oil to prevent evaporation. To ensure reliability, each LAMP reaction included a template and non-template control. A custom fluorescent threshold-based algorithm was used to track the amplification curve for each sample and determine the corresponding amplification times.

**Figure 1:**
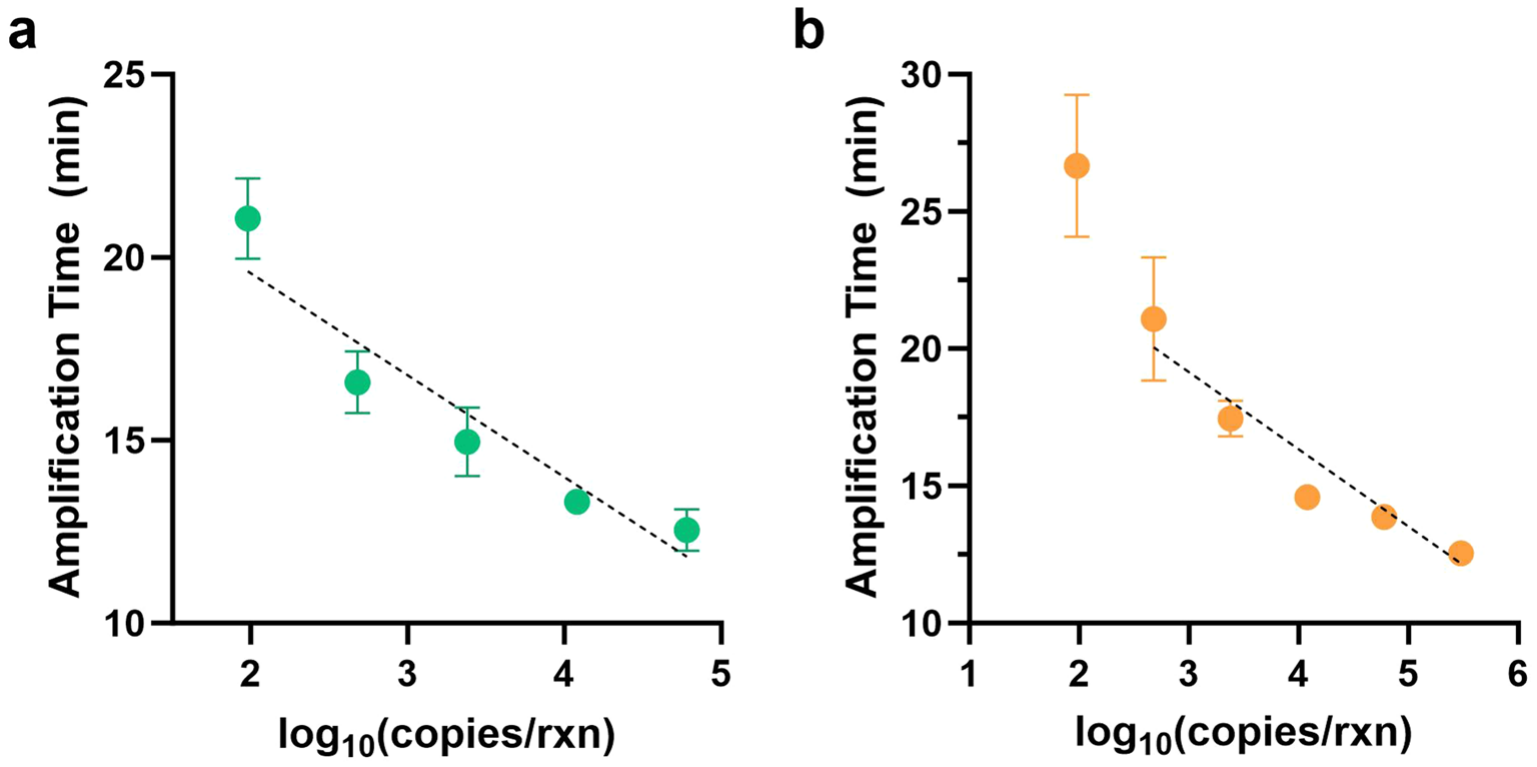
Quantitative LAMP assays. (**a**) The calibration for the human GAPDH assay extended from approximately 60,000 to 96 copies per reaction, r^2^ = 0.92 (n = 4). (**b**) The calibration curve for the KSHV ORF26 assay was limited to the linear range of approximately 300,000 to 480 copies per reaction, r^2^ = 0.93 (n = 2).

For quantitative LAMP results, calibration curves were generated for each LAMP assay. The concentrations of human DNA extractions and ORF26 plasmid stock solutions were quantified spectroscopically (QuickDrop, SpectraMax) before serial dilutions were performed. The human GAPDH calibration (Fig. 1a) extended from 60,000 to 96 copies per reaction (r^2^ = 0.92) while the KSHV ORF26 calibration (Fig. 1b) was limited to the linear region from 300,000 to 480 copies per reaction (r^2^ = 0.93). All statistical analyses, including the calibration curve fits and unpaired student t-tests to compare DNA yields, were performed in Prism (GraphPad).

### DNA extractions

#### Alkaline extraction

The alkaline extraction, commercially available as HotSHOT, is Hot Sodium HydrOxide and Tris: a 30-min incubation at 95 °C in a sodium hydroxide solution followed by the addition of a neutralizing buffer^18,19^. As a consideration for POC applications, the protocols were adjusted to eliminate heat during the incubation; thus, the Sodium HydrOxide and Tris without heat (ColdSHOT) DNA extraction was performed at ambient temperature (23 °C). The 0.75 mm skin punch biopsies were incubated in 25 mM NaOH followed by neutralization with an equal volume of 100 mM Tris-HCL and 0.5 mM EDTA. The 25 mM NaOH solution was prepared from a 1 M NaOH solution (Sigma Aldrich 1310-73-2) while the neutralizing solution was prepared from 0.5 M Tris-HCl (pH 8, Thermo Fisher J67510.AE) and TE buffer (pH 8, Promega V6231). All necessary dilutions were performed using DEPC-treated water (Invitrogen, AM9915G).

#### Silica-based spin column extraction

The yield of the ColdSHOT DNA extraction was compared to that of a standard silica-based spin column extraction (DNeasy Blood and Tissue Kit, QIAGEN 69504). Briefly, tissue samples were incubated in 20 μl of proteinase K and 180 μl of ATL buffer at 56 °C. Once clear, 200 μl of AL buffer and 200 μl of ethanol were added, with intermediate vortexing. DNA purification was performed via two wash steps using spin columns and the provided washing buffers. Finally, the DNA was eluted from the spin column membrane into AE buffer. The volume of AE buffer was adjusted from the manufacturer’s protocol to match the volume of the ColdSHOT DNA extraction.

### KSHV ORF26 plasmid spike experiments

ORF26 plasmids^29^ were spiked into ColdSHOT extractions with KS-negative skin sample to determine whether varying amounts of non-template tissue might affect KSHV viral load quantification. The amount of tissue varied from 1.5 to 15 mg while the copies of ORF26 added remained constant at ~ 20,000 copies per reaction.

### Sample storage experiments

Storage experiments were performed to mimic the storage and transfer of patient samples. Tissue samples were placed in PCR tubes and immersed in 200 µl of either RNA Later (Thermo Fisher, AM7020), DNA Shield (Zymo Research, R1100), TE buffer (pH 8, Promega, V6231), or PBS (pH 7.4, Thermo Fisher, 10010023). The samples were stored at either 23 °C or − 20 °C for 48 h (n = 4). After 48 h, the storage solutions were removed with a pipette, and the DNA was extracted using ColdSHOT and quantified. In separate experiments, DNA extracts after ColdSHOT were stored at either 23 °C or − 20 °C (n = 4). At one-week intervals spanning four weeks, 5 µl of each sample were tested to assess the stability of the extracted DNA to both prolonged storage at 23 °C and several freeze–thaw cycles.

### Varying NaOH concentrations and addition of pH indicators

The robustness of ColdSHOT yields was assessed across varying initial NaOH concentrations, and the addition of pH indicators was evaluated to provide clear visual feedback of extraction conditions. The starting NaOH solutions evaluated included 1, 10, 25, 50, and 100 mM—all prepared from a 1 M NaOH solution (Sigma Aldrich 1310-73-2) and diluted using DEPC-treated water (Invitrogen, AM9915G). The pH indicators considered were (1) Thymolphthalein (Thermo Fisher B23896.09), (2) O-cresolphthalein (Sigma Aldrich C85778), and (3) Alizarin Yellow R (Thermo Fisher 038707.09). Stock solutions of the dyes were prepared in 96% ethanol (Sigma Aldrich 1590100500) at 10 mg/ml for thymol- and O-cresol phthalein and 5 mg/ml for Alizarin Yellow R. The indicator solutions were added to the NaOH solutions at 1% by volume.

## Results

### Optimization of the ColdSHOT DNA extraction

**Figure 2:**
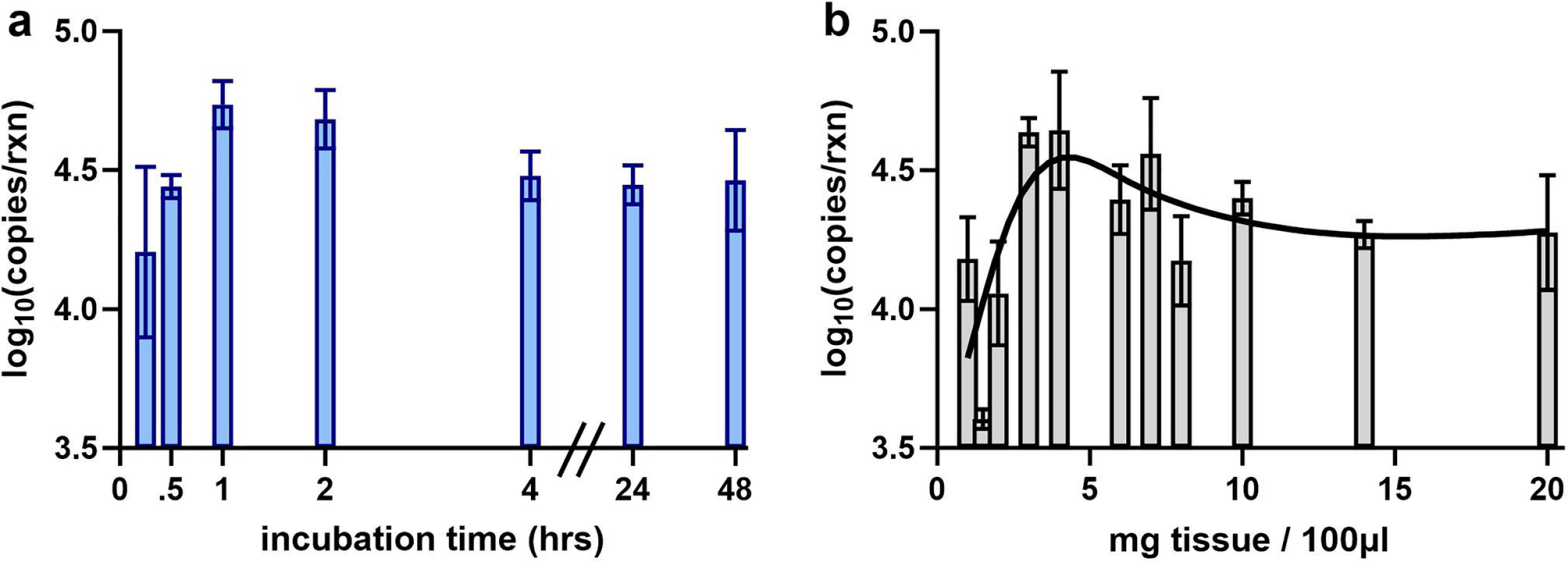
Optimizing the ColdSHOT DNA extraction. (**a**) The submillimeter tissue biopsies were incubated in 25 mM NaOH solution for varying timepoints and a peak DNA yield was observed after a 1-h incubation (n = 4). (**b**) The optimal ratio of tissue mass to extraction volume was found to be approximately 4 mg of tissue per 100 µl of solution after a spline fit of the data.

The optimal incubation time for the ColdSHOT DNA extraction was determined using 3 mg skin tissue samples in a final volume of 75 µl. The incubation periods in 25 mM NaOH included 10, 30, and 60 min as well as 2, 4, 24, and 48 h (Fig. 2a). An increasing DNA yield from 10 to 60 min was observed with a maximum yield of ~ 50,000 copies per reaction (log-scale: µ = 4.74, SD = 0.09, n = 4). After 60 min, the yield plateaued, suggesting the ColdSHOT yield was robust to modest variations in the incubation time. For POC applications, the 1-h extraction process would facilitate faster turnaround times than those currently achievable using conventional spin column extractions.

Following the time optimization, the optimal ratio of skin tissue to solution volume was determined for a 1-h incubation. Tissue masses ranged from 1.5 to 15 mg while extraction volumes ranged from 75 to 200 µl. The DNA yields of each mass-to-volume trial, normalized to milligrams of tissue per 100 µl extraction volume, were fit with a spline curve (Fig. 2b). At low mass-to-volume ratios (< 3 mg/100 µl), DNA yields were smaller and more variable. Around 4 mg of tissue per 100 µl, a peak DNA yield of ~ 45,000 copies per reaction was observed (log-scale: µ = 4.64, SD = 0.21, n = 7). With higher tissue to volume ratios, the DNA yields decreased slightly and plateaued. While additional tissue would presumably lead to more DNA, a possible explanation for the plateau is an imbalance of DNA copies and mastermix reagents; previous work with RT-LAMP demonstrated amplification inhibition with high amounts of RNA^30^. For the 20 µl GAPDH LAMP assay used in this study, a ratio of 4 mg of tissue per 100 µl of extraction volume produced the highest DNA yields.

### Assessment of the ColdSHOT DNA extraction yield

**Figure 3:**
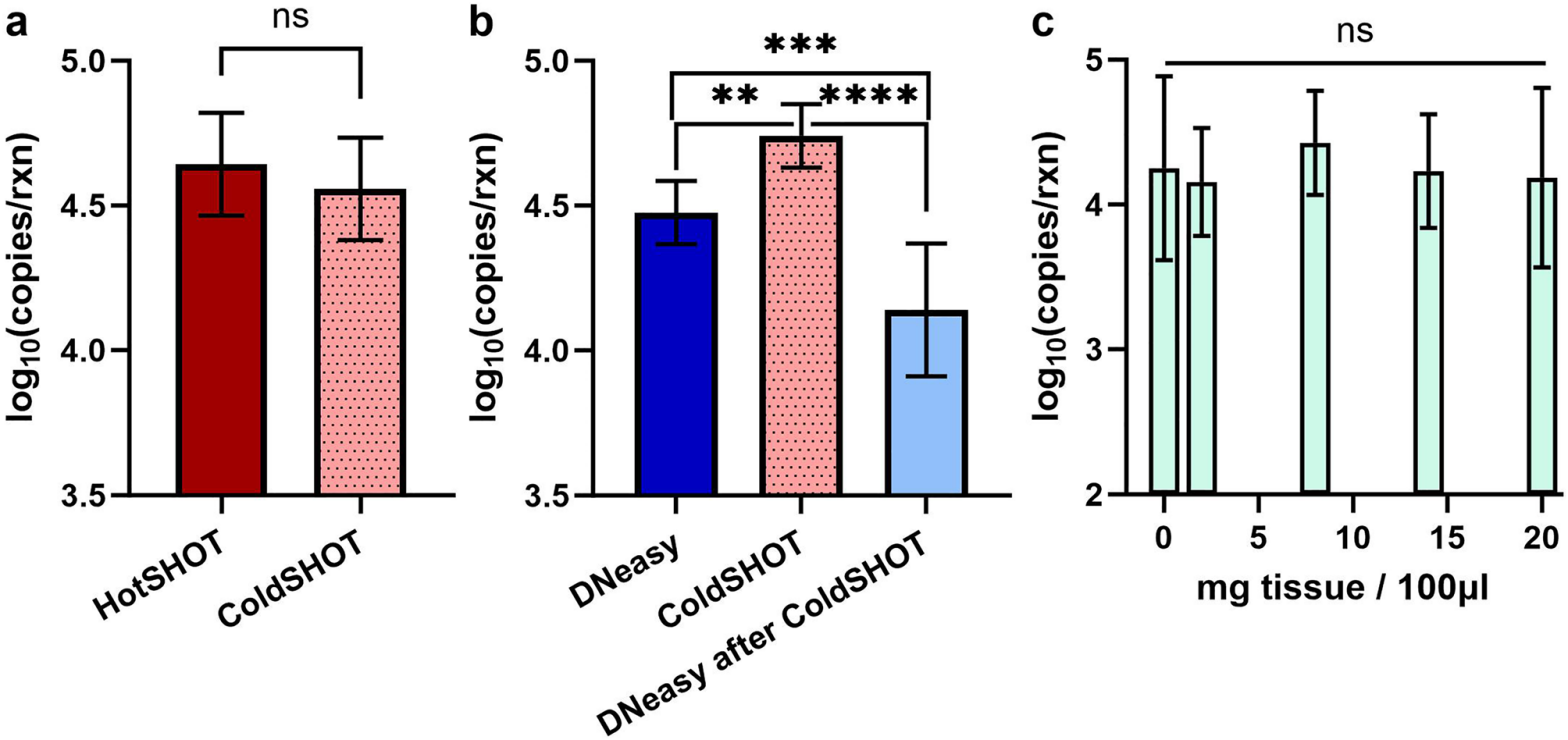
Analysis of the ColdSHOT DNA yield. (**a**) No difference in DNA yield was observed between HotSHOT (30-min incubation at 95 °C) and ColdSHOT (1-h incubation at 23 °C) (n = 7). (**b**) ColdSHOT provided more DNA copies than the DNeasy spin column extraction (p = 0.0003, n = 8). Using the DNeasy extraction on the tissue fragments remaining after ColdSHOT resulted in significantly fewer DNA copies compared to DNeasy (p = 0.002, n = 8) and ColdSHOT (p < 0.0001, n = 8). (**c**) When quantifying KSHV plasmids spiked into ColdSHOT extractions, the quantity of non-target tissue did not impact the expected yield (n = 3).

DNA yields were quantified for the ColdSHOT (1-h ambient incubation) and HotSHOT (30-min incubation at 95 °C) extractions (Fig. 3a). Each experiment utilized 3 mg tissue samples and 75 µl extraction volumes (n = 7). The log-scale DNA copies per reaction recovered by HotSHOT (µ = 4.65, SD = 0.18) were not significantly different from those of ColdSHOT (µ = 4.56, SD = 0.18), and the methods yielded ~ 44,000 and ~ 36,000 copies per reaction, respectively.

For comparison to a laboratory standard DNA extraction, ColdSHOT was compared to a silica membrane spin column extraction (DNeasy Blood and Tissue Kit). For each trial, 3 mg skin tissue samples were extracted into 75 µl (Fig. 3b). Significantly more DNA (p = 0.0003) was recovered with ColdSHOT (log-scale: µ = 4.74, SD = 0.11, n = 8) than with DNeasy (log-scale: µ = 4.48, SD = 0.11, n = 8), and the methods yielded ~ 55,000 and ~ 30,000 DNA copies per reaction, respectively. As ColdSHOT did not fully digest tissue samples, the DNA remaining in the tissue fragments was recovered by an additional DNeasy extraction after ColdSHOT (Fig. 3b). After ColdSHOT, the DNA yield from the tissue fragments was reduced to ~ 14,000 copies per reaction (log-scale: µ = 4.14, SD = 0.23, n = 8), a significant reduction as compared to the original ColdSHOT yield (p < 0.0001) and original DNeasy yield (p = 0.002).

To determine whether the amount of tissue in a sample would affect KSHV detection, ORF26 plasmids were spiked into ColdSHOT extractions as a proxy. The number of ORF26 copies was kept constant at ~ 20,000 copies per reaction while the amount of tissue ranged from 1.5 mg to 15 mg per 75 µl extraction volume (n = 3). For all groups, no significant difference in DNA yields was observed, indicating that the LAMP assay was not affected by the quantity of non-template cellular debris present in the sample (Fig. 3c).

These experiments demonstrate ColdSHOT’s compatibility with POC applications and comparability to previously established methods. In settings where the ambient temperature exceeds 23 °C, the congruence in yields between HotSHOT at 95 °C and ColdSHOT at 23 °C indicates ColdSHOT will still be effective. The DNA yields for the ColdSHOT and DNeasy spin column extractions were approximately 1 µg of DNA per milligram of tissue—a yield similar to Qiagen’s estimate and empirical observations^31^. Though ColdSHOT does not fully digest solid tissue, the remaining tissue fragments appeared noticeably larger and had significantly less DNA, indicating some level of tissue penetration by the NaOH. Additionally, the LAMP-based quantification of the KSHV target sequence was independent of the quantity of KS-negative tissue in the sample. Thus, these results indicate ColdSHOT can provide similar DNA yields to the laboratory standard in less time and fewer steps without significant equipment.

For POC KS diagnosis, the yields of the ColdSHOT extraction demonstrate potential for clinical accuracy. In previous work, the LAMP-based KSHV viral load used to differentiate KS-positive and KS-negative samples from patients in Uganda was ~ 96 KSHV copies per reaction^9^. From PCR analysis of 17 KS lesions, a median of 0.67 viral copies per cell was reported^32^. ColdSHOT consistently yielded ~ 40,000 GAPDH DNA copies, or the DNA from ~ 20,000 cells, per reaction. Combining a conservative ColdSHOT yield of ~ 10,000 cells’ worth of DNA with ~ 0.67 KSHV copies per cell would result in ~ 6,700 KSHV copies per reaction which is sufficient based on the 96-copy threshold reported^9^. However, for diagnostic applications, it is important to consider the heterogeneity of KS lesions. Image analysis of histopathology slides revealed only an average of 49% of cells stained for KSHV-encoded latency-associated nuclear antigen^32^. Thus, using submillimeter biopsies would require careful sampling of the KS lesion to minimize false-negative results due to sampling error. Furthermore, it is imperative that the primer set(s) used to detect KSHV in patient samples maintain(s) sensitivity despite any genetic variations across KS subtypes and/or mutations found in KS lesions^33,34^. With a robust LAMP assay and sufficient sample, these results indicate the ColdSHOT DNA yields are sufficient for LAMP-based KS diagnosis.

### Sample storage experiments

**Figure 4:**
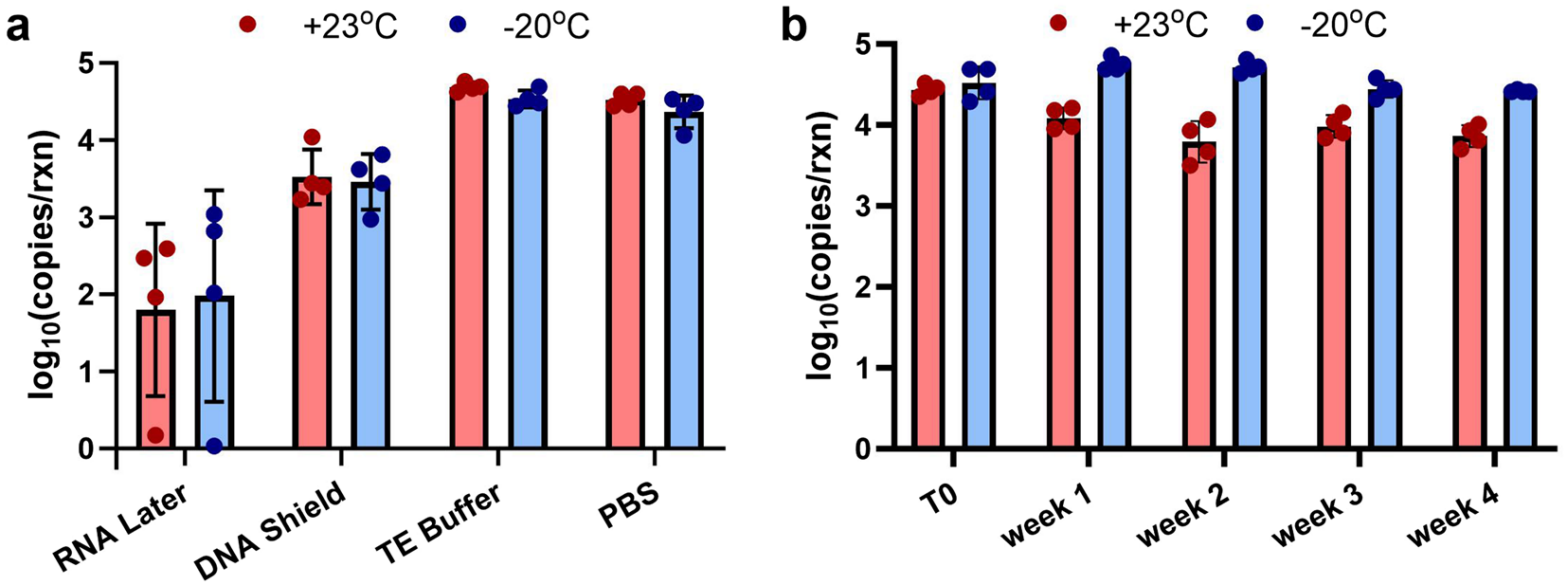
Robustness of ColdSHOT DNA extraction to storage conditions. (**a**) Prior to ColdSHOT extraction, storage in commercial buffers RNA Later and DNA Shield resulted in lower DNA yields while storage in TE buffer and PBS maintained the expected yields (n = 4). (**b**) After ColdSHOT extraction, 23 °C storage conditions resulted in a decrease in DNA yield while samples stored frozen maintained their yield after 4 weeks and 4 freeze–thaw cycles (n = 4).

To assess the stability of the ColdSHOT DNA yields to storage, tissue samples were stored before ColdSHOT, and neutralized DNA solutions were stored after extraction. Before extractions, tissue samples were stored in various solutions for 48 h at either 23 °C or − 20 °C (n = 4). Storage in TE buffer and PBS demonstrated good retention of the original DNA yield while storage in RNA Later and DNA Shield reduced the DNA yields (Fig. 4a). After ColdSHOT, the neutralized solutions containing extracted DNA were stored at 23 °C and − 20 °C (n = 4). Samples stored at − 20 °C demonstrated consistent results through 4 weeks and several freeze–thaw cycles (Fig. 4b). While a decrease in the DNA copy numbers was observed for the 23 °C group, all stored samples consistently amplified after 4 weeks.

These results demonstrate good compatibility of the ColdSHOT extraction with typical sample storage protocols. For tissue storage before ColdSHOT, the reduced yields with commercial storage buffers RNA Later and DNA Shield are consistent with previous findings^16,20,35,36^ and may be explained by the commercial products permeating the tissue and developing a “hard, rubbery texture” which “may be more difficult to homogenize thoroughly” (RNAlater product information, Sigma-Aldrich). Thus, where immediate sample processing is not possible, samples should be stored in either TE buffer or PBS. For storage both before and after extraction, samples should ideally be stored at − 20 °C, though these results indicate the feasibility of some ambient storage up to 23 °C. For applications with ambient temperatures greater than 23 °C, further work should be done to verify sample viability after prolonged ambient storage. Overall, these results demonstrate the practicality of the ColdSHOT DNA extraction for POC applications as samples may be stored for later analysis and/or any necessary sample reruns.

### Varying NaOH concentrations and addition of pH indicators

**Figure 5:**
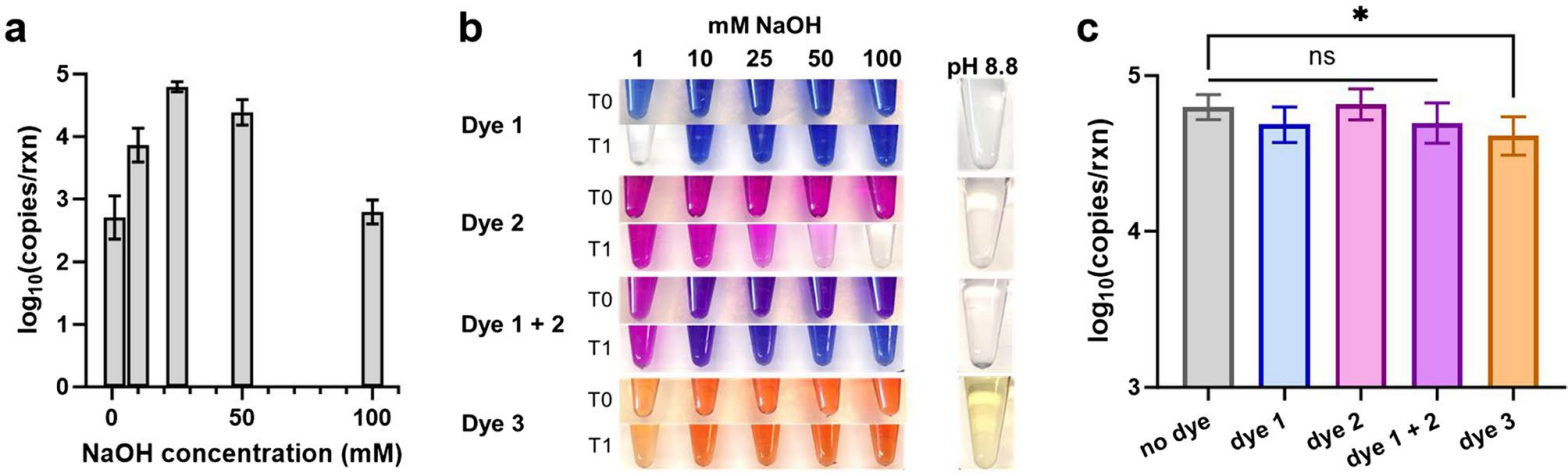
ColdSHOT yields with varying NaOH concentration and added pH indicator dyes. (**a**) The peak DNA yield for ColdSHOT was observed with a 25 mM NaOH concentration (n = 4). (**b**) The indicator dyes considered were 1) Thymolphthalein, 2) O-cresolphthalein, and 3) Alizarin Yellow R. From the initial time (T0), some color changes were observed after storage for three weeks (T1). (**c**) Compared to a no-dye control, adding the indicator dye solutions (1% by volume) to the NaOH solution demonstrated no significant difference in the ColdSHOT DNA yield except in the case of Alizarin Yellow (p = 0.045, n = 4).

The ColdSHOT extraction relies on alkalinity to extract DNA by hydrolyzing cellular and nuclear membranes and the subsequent neutralization to ensure the stability of the product and suitability for amplification^18–20,25^. When comparing NaOH solutions with concentrations ranging from 1-100 mM, the 25 mM NaOH produced the highest DNA yield at ~ 50,000 copies per reaction (Fig. 5a). Lower NaOH concentrations of 10 mM and 1 mM resulted in smaller yields of ~ 7,000 and ~ 500 copies per reaction, respectively. Higher NaOH concentrations of 50 mM and 100 mM also resulted in smaller yields of ~ 25,000 and ~ 500 copies per reaction, respectively. The lower yields observed with higher NaOH concentrations were likely the result of slight amplification inhibition by excess sodium ions as similar results have been reported during extractions from cell lysates^20^. Additionally, other applications of the alkaline extraction use up to 0.5 M NaOH but require larger ratios of neutralizing buffer^37–39^.

As the anticipated failure modes of the ColdSHOT DNA extraction involve improper pH conditions, various pH indicators were assessed. The ideal indicator would clearly identify the NaOH lysis solution (pH ~ 12.4) and indicate when the ColdSHOT reaction was sufficiently neutralized (pH < 9) without affecting the DNA yields. Thymolphthalein, O-cresolphthalein, and their combination each satisfied these requirements by providing a strong color in 25 mM NaOH, turning colorless after neutralization, and not significantly affecting DNA yields (Fig. 5b, c). While Alizarin Yellow provided a subtle gradient, it resulted in a slight loss of DNA yield, possibly due to added sodium ions slowing the amplification. For instances when the indicators would be added well in advance of ColdSHOT, the shelf-life of the indicators needs further investigation as fading was observed after 3 weeks. After the fading, the combination of the Thymolphthalein and O-cresolphthalein provided the best gradient as the 25 mM NaOH was blue while weaker NaOH solutions were slightly purple or pink. These results demonstrate the feasibility of adding simple pH indicators to clearly identify the NaOH lysis buffer and indicate sufficient neutralization of the extraction.

## Discussion and conclusion

In this study, a simplified, POC-compatible alkaline DNA extraction—ColdSHOT—was optimized for 0.75 mm human skin punch microbiopsies and LAMP-based testing. For this equipment-free extraction, the optimal conditions were determined as follows: 1-h ambient incubation in 25 mM NaOH followed by the addition of an equal volume of 100 mM Tris and 0.5 mM EDTA. Peak DNA yields, quantified via real-time LAMP, were observed at a tissue-to-volume ratio of 4 mg-to-100 µl final volume.

Compared to traditional spin column membrane extractions, ColdSHOT has many advantages for POC applications, though several contraindications must be considered. Using the conditions explained previously, ColdSHOT provides similar DNA yields to spin column extractions in an hour without centrifugations or heated incubations. ColdSHOT requires only two reagents and a pipette, thus it is a rapid, affordable, and easy-to-use DNA extraction method. However, not all applications and sample types will be suitable for ColdSHOT. As no purification steps are included, crude DNA samples could interfere with nucleic acid amplification. While this study observed no inhibition of the LAMP reaction with crude skin samples, other sample types and applications requiring PCR may necessitate further sample dilution or purification. Additionally, for solid tissue samples, sample size is an important factor as ColdSHOT does not fully digest tissues; DNA was effectively extracted from 0.75 mm punch biopsies, but ColdSHOT may not effectively penetrate larger tissue samples and could leave significant DNA in the undigested tissue bulk. Despite these limitations, ColdSHOT remains a promising DNA extraction for LAMP-based applications using small tissue samples without known inhibitors.

For the POC detection of KSHV in skin punch biopsies, ColdSHOT is a promising DNA extraction method. This study demonstrates that ColdSHOT is a rapid, easy, and affordable means to extract DNA from submillimeter skin biopsies without significant equipment. Further, the extraction is robust to sample storage before and after extraction. In conclusion, these results indicate that using the ColdSHOT DNA extraction on submillimeter biopsy samples could simplify and accelerate LAMP-based diagnosis of Kaposi’s sarcoma.

### Supplementary Information

Supplementary Tables.

## Data Availability

The datasets generated are available upon request from the corresponding author.
