# Supplementary material for: Rapid, equipment-free extraction of DNA from skin biopsies for point-of-care diagnostics
Source: Sci Rep. 2024 Jun 14;14:13782. doi: 10.1038/s41598-024-64533-3 (PMC11178891; doi:10.1038/s41598-024-64533-3)
Supplement: Supplementary file 1 — Supplementary Tables. [file 41598_2024_64533_MOESM1_ESM.pdf]

Rapid, equipment-free extraction of DNA from skin biopsies for point-of-care diagnostics.

Manning, Jason Cade[1]; Boza, Juan Manuel[1]; Cesarman, Ethel[2]; Erickson, David[3,4*]

[1]Meinig School of Biomedical Engineering, Cornell University; Ithaca, NY 14850, USA
[2]Pathology and Laboratory Medicine, Weill Cornell Medical College; New York, NY, 10021, USA.
[3]Sibley School of Mechanical and Aerospace Engineering, Cornell University; Ithaca, NY 14850, USA
[4]Division of Nutritional Sciences, Cornell University; Ithaca, NY 14850, USA

***Corresponding Author:** David Erickson, PhD, Cornell University, 369 Upson Hall, Cornell University, Ithaca, NY 14853, Tel: 607-342-1799, Fax: 607-255-1222, Email: de54@cornell.edu

Supplementary Table S1: Primer sequence for human GAPDH LAMP assay

Primer	Sequence (5' > 3')	Concentration (μM)
F3	TGGGGAATGGGACTGAGG	0.4
B3	GTGGCAGTGATGGCATGG	0.4
FIP	TGGGAAAGCCAGTCCCCAGAACTCATCCAAGACTGGCTCCT	1.6
BIP	CTTTCAAGGTGGGGAGGGAGGACTGTGGTCTGCAAAAGGAG	1.6
LF	AACCCCAGGGTTGCACG	0.8
LB	TAGAGGGGTGATGTGGGGAGTA	0.8

Supplementary Table S2: Primer sequence for KSHV ORF26 LAMP assay

Primer	Sequence (5' > 3')	Concentration (μM)
F3	TGCCCCCTTTTTTCAGTGG	0.4
B3	CCGGCCGATATTTTGGAGT	0.4
FIP	TGGATTCGAGCACAATGGTGGACAACACCCAGCTAGCAGTG	1.6
BIP	TCGTGTTCCCCATGGTCGTGAGATGTGGTACACCAACAGC	1.6
LF	CCTTTCGGCTAAAAAATGGGGGTAG	0.8
LB	CAGCAACTGGGGCACGCTAT	0.8

Supplementary Table S3: LAMP assay mastermix

Reagent	GAPDH 1X (μL)	KSHV ORF26 1X (uL)	Source
Isothermal Buffer (10X)	2.5	4.0	NEB B0537S
$MgSO_4$ (100 mM)	1.5	2.4	NEB B1003S
dNTPs (10 mM)	3.5	5.6	NEB N0447L
25X Primer Mix	1	1.6	IDT Technologies
Bst 2.0 WarmStart (8,000U/mL)	1	1.6	NEB M0538L
Evagreen (20X)	1.25	2.0	Biotium 31000
Rox Reference Dye (50X)	0.5	1.6	Thermo Fisher 12223012
Nuclease-free water	8.75	16.2	Invitrogen AM9915G
Total before 5μl sample:	20.0	35.0	
